# Factors impacting hospital avoidance program utilisation in the care of acutely unwell residential aged care facility residents

**DOI:** 10.1186/s12913-021-06575-1

**Published:** 2021-06-24

**Authors:** Luke Testa, Tayhla Ryder, Jeffrey Braithwaite, Rebecca J. Mitchell

**Affiliations:** Australian Institute of Health Innovation, Macquarie University, Level 6, 75 Talavera Road, Sydney, NSW 2109 Australia

**Keywords:** Aged, Aged, 80 and over, COVID-19, Health Services for the Aged, Nursing homes, Patient care team, Patient transfer, Referral and consultation

## Abstract

**Background:**

An existing hospital avoidance program, the Aged Care Rapid Response Team (ARRT), rapidly delivers geriatric outreach services to acutely unwell or older people with declining health at risk of hospitalisation. The aim of the current study was to explore health professionals’ perspectives on the factors impacting ARRT utilisation in the care of acutely unwell residential aged care facility residents.

**Methods:**

Semi-structured interviews were conducted with two Geriatricians, two ARRT Clinical Nurse Consultants, an ED-based Clinical Nurse Specialist, and an Extended Care Paramedic. Interview questions elicited views on key factors regarding care decisions and care transitions for acutely unwell residential aged care facility residents. Thematic analysis was undertaken to identify themes and sub-themes from interviews.

**Results:**

Analysis of interviews identified five overarching themes affecting ARRT utilisation in the care of acutely unwell residents: (1) resident care needs; (2) family factors; (3) enabling factors; (4) barriers; and (5) adaptability and responsiveness to the COVID-19 pandemic.

**Conclusion:**

Various factors impact on hospital avoidance program utilisation in the care of acutely unwell older aged care facility residents. This information provides additional context to existing quantitative evaluations of hospital avoidance programs, as well as informing the design of future hospital avoidance programs.

**Supplementary Information:**

The online version contains supplementary material available at 10.1186/s12913-021-06575-1.

## Introduction

Population ageing is occurring in many countries, including Australia [1]. Ageing is associated with multiple chronic health conditions and greater use of health and aged care services [2, 3]. Rising hospital service utilisation has been observed, with older people overrepresented in the hospitalised population [4]. Residential aged care facility (RACF) residents (also known as nursing home residents) are a vulnerable population that experience greater challenges and poorer outcomes when transferring from the RACF to Emergency Departments (EDs) [5].

Care for acutely unwell RACF residents for some health conditions can potentially be delivered outside of an acute hospital setting [6]. It has been previously estimated that 5–60% of resident transfers to EDs are avoidable [3]. Hospital avoidance programs have been designed and implemented in multiple aged care settings to reduce unnecessary ED transfers and the associated risks, such as care complications [7–10]. A variety of approaches to reducing avoidable hospital admissions have been used, with mixed results [11].

An existing hospital avoidance program, the Aged Care Rapid Response Team (ARRT), rapidly delivers geriatric outreach services to older people in declining health or acutely unwell and at risk of hospitalisation [12]. Previously, analysis of data collected by ARRT over a three year period indicated that residents were successfully managed within their RACF on 85% of occasions and hospital transfer was potentially avoided on almost half of occasions [12]. Additionally, the effect of ARRT on health service utilisation and resident health outcomes found that residents reviewed by ARRT who were subsequently hospitalised had an average reduction of 9–10 days in hospital length of stay and AUD$2000–$8000 in hospital treatment costs compared to residents receiving usual care [12].

The ARRT model of care is complex and contains multiple service elements, including a geriatric outreach service, coordination of resident care, and capability building of RACF staff. Identifying the elements that influence resident outcomes will aid in future hospital avoidance model design and subsequent implementation. The current study aims to explore health professionals’ perspectives on the factors impacting ARRT utilisation in the care of acutely unwell residential aged care residents.

## Methods

### Design

Semi-structured interviews of health professionals working in the ARRT health service area were conducted. Two theoretical frameworks informed the approach: Donabedian’s model [13] and the modified Andersen’s health behavior model [14]. Donabedian’s model provides a framework for evaluating health services using three separate but interrelated concepts: structure, process and outcome. Structure describes factors influencing the setting in which ARRT delivers care. e.g. physical, organisational and staffing characteristics. Process refers to all activities undertaken by ARRT that deliver and support resident care. Outcome refers to the effect of ARRT processes on residents, health professionals and the health system. Structures, processes and outcomes relating to ARRT were identified from reviewing the ARRT model of care and relevant literature [11, 15, 16].

**Fig. 1:**
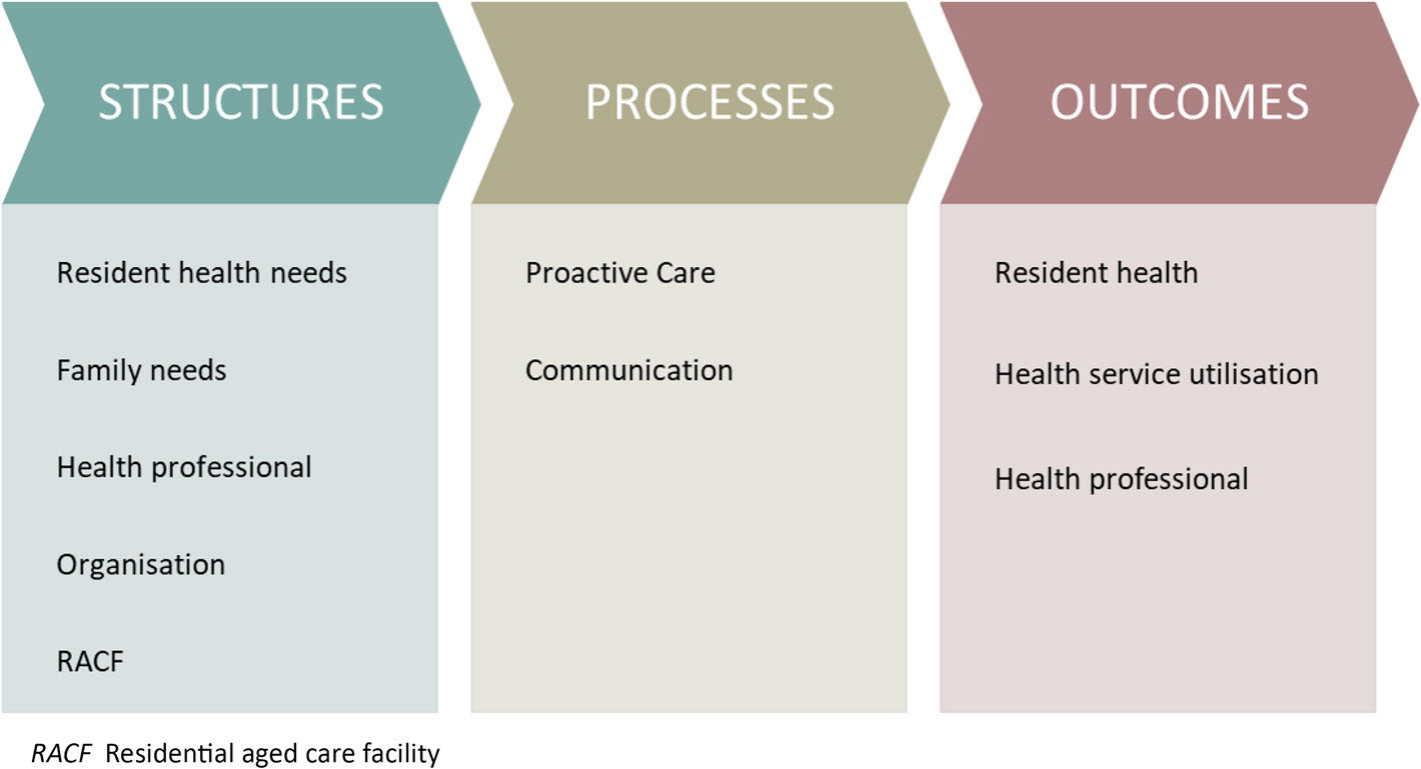
Structures, processes and outcomes of the Aged Care Rapid Response Team. RACF: Residential aged care facility

Andersen’s health behavior model depicts health service utilisation as a consequence of three broad categories: need factors, predisposing factors and enabling factors. Previously, Andersen’s health behavior model was adapted for the ED setting [17] and has been modified further for ED utilisation by older adults [14]. The modified Andersen’s health behavior model conceptualises the unwell older person’s need for ED transfer from a RACF in relation to the intensity of their health care needs (i.e. the predisposing factor). Enabling factors were conceptualised as those preventing ED transfers. The organisation of residents’ health care needs in tandem with their need for ED transfer and enabling factors allowed the modified Andersen’s health behavior model to be envisioned as a subset of Donabedian’s model (Fig. 1). A priori categories relating to structures, processes and outcomes were used to guide the interview questions and analysis (Table 1)

**Table 1:** A priori categories for structure, process and outcome elements of the Aged Care Rapid Response Team

Category	Definition
**STRUCTURES**	
**Resident health needs**	The necessity of ED transfer and/or hospital admission when an RACF resident becomes unwell
Comorbidity	The presence of two or more co-occurring medical conditions
Functional decline	The loss of physical and/or cognitive abilities
End of life	RACF residents at the end stage of life
**Family needs**	Needs and expectations of the family of an unwell RACF resident
**Health professional**	ARRT team roles (Geriatrician, Clinical Nurse Consultants, Aged Care Registrar)
Experience	Experience required by ARRT staff to perform their role
Knowledge	Knowledge required by ARRT staff to perform their role
Skills	Skills required by ARRT staff to perform their role
**Organisation**	Physical and organisational factors required to deliver ARRT
Setting	Program description and characteristics
Resources	Resources required by ARRT to operate. Includes access to technology.
Workload	Amount/difficulty of work assigned to ARRT staff
Barriers	Challenges in delivering ARRT
Enablers	Facilitators for delivering ARRT
**Residential Aged Care Facility**	RACF staff roles
Training	Training required by RACF staff roles to perform their role
Knowledge	Knowledge required by RACF staff to perform their role
Skills	Skills required by RACF staff to perform their role
**PROCESSES**	
**Proactive Care**	A person-centred, preventative approach to the care of unwell RACF residents
Access to skilled care providers	Rapid access to appropriate decision making and care. Includes capacity to access additional expertise e.g. nurse practitioners, allied health practitioners, pharmacists, geriatricians, palliative care specialists, medicolegal and referrals for specialist services
Coordinated care	Delivering care that is integrated between multiple providers and services
CGA	An assessment of medical, social, and functional needs, and the development of a coordinated care
Advance Care Planning	Resident’s preferences for future care
Capability building of RACF staff	Improving care through sharing knowledge and skills with RACF staff
Risk stratification	Identification of RACF residents who are at risk of hospitalisation and likely to benefit from ARRT intervention
Partnerships	Hospitals, RACFs, ambulance services and GPs working together to achieve shared goals
**Communication**	Exchange of information regarding the care of RACF residents
Interpersonal communication	Sharing of information between health professionals, RACF residents and the families via personal interactions
Information transfer	The handover of resident health data from one care provider to another
**OUTCOMES**	
**Resident health**	Effect of ARRT on health of unwell RACF residents
Resident managed in RACF	Factors involved in RACF-based management of unwell RACF resident
Resident transferred to ED	Factors involved in ED transfer of unwell RACF residents
Resident admitted to hospital	Factors involved in hospital admission of unwell RACF residents
Adverse events	Adverse events associated with ED transfer/hospital admission of unwell RACF residents
Health system utilisation	Effect of ARRT on the use of hospital services by unwell RACF residents
Health professional	Effect of ARRT on health professionals involved in the care of unwell RACF residents
**ADAPTABILITY AND RESPONSIVENESS**	ARRT’s ability to adapt to changing circumstances e.g. pandemics/disasters
COVID-19	All COVID-19 information

*CGA* Comprehensive Geriatric Assessment, *ED* Emergency Department, *RACF* Residential aged care facility

.

Ethical approval for this evaluation was granted by the Northern Sydney Local Health District Human Research Ethics Committee (reference: RESP/18/247) and Macquarie University Human Research Ethics Committee (reference: 5201938347115).

Adapted from Donabedian’s model [13] and a modified Andersen’s health behavior model [14].

### Setting

ARRT is a hospital-based service operating in Northern Sydney Local Health District (NSLHD), Australia. As of 30th June 2015, NSLHD had a population of approximately 900,000, with an estimated 15.5% aged 65 years and older [18]. ARRT supports home and RACF-based management of older people at risk of hospitalisation, when appropriate, through the rapid delivery of outreach services to the Lower North Shore and Ryde/Hunters Hill regions of NSLHD. The ARRT service area encompasses 43 local RACFs (3344 RACF beds in total) and two public hospitals (a 720 bed and a 160 bed hospital). ARRT operates from Monday to Friday from 8 am to 4.30 pm and is staffed by a hospital-based geriatrician, an aged care community registrar and two Clinical Nurse Consultants (CNCs). Referrals can be made to ARRT via telephone. There is a dedicated phone line for the ARRT service, as well as direct contact numbers for the CNCs and registrar for urgent referrals. Upon referral by RACF staff, the ARRT CNC/Registrar assess and triage the resident. Telehealth may be used to support the assessment and triage process. If a RACF visit is required, it will usually occur within 24 h. Services provided by ARRT include: Comprehensive Geriatric Assessment, treatment of acute infections, liaison with other services e.g., NSW ambulance, mobile x-ray and ultrasound, falls assessment, pain management and medication review [19].

### Participants

Purposive sampling [20] was used to identify a representative sample of health professionals involved in the care of unwell RACF residents, with the assistance of the ARRT Geriatrician. Ten potential participants were invited to join the study via email. This included two Geriatricians, two Aged Care Registrars, two ARRT CNCs, one ED-based Clinical Nurse Specialist, one Extended Care Paramedic, one General Practitioner, and one RACF manager. If no response was received, a follow-up email was sent. Six participants consented to be interviewed and four did not respond. A date and time to be interviewed was organised at the discretion of the consenting participants. Participants were emailed an interview guide prior to their interview date. Participants were interviewed individually by LT.

### Data collection

LT conducted semi-structured interviews via Zoom video conferencing in October 2020. At the time of the study, LT was a PhD candidate and Research Assistant with postgraduate qualifications in public health and research, and experience in collecting and analysing data from interviews and focus groups. Prior to the current study, LT co-authored two ARRT evaluation studies with two of the current study’s participants. Before commencing interviews, all participants were informed that the current study formed part of LT’s PhD. Interviews took between 15 and 70 min to complete. Each interview was audio recorded and transcribed verbatim. Notes were taken during and/or after interviews. Interview questions were asked to elicit descriptions on key factors regarding care decisions and care transitions for acutely unwell RACF residents, as well as the structures, processes and outcomes of the ARRT model of care (Appendix A). Staff outcomes were assessed using a single-item job satisfaction measure [21] with an open-ended follow-up question.

### Data analysis

Interview transcripts were imported to NVivo 12 [22] for coding and thematic analysis. A list of preliminary codes based on the a priori categories was created prior data collection. A coding system was adopted to encourage intercoder reliability and agreement [23]. An interview transcript was selected at random and dual coded by LT and TR. Coding was compared and discrepancies were discussed. Refinements to codes and definitions were suggested and mutually agreed upon. Where LT and TR could not reach agreement, RM provided guidance. The dual coding process was repeated two times until LT and TR were satisfied with modifications to the code list and the level of intercoder agreement. The remaining transcripts were then divided and coded individually.

Once coding was complete, data were charted into a framework matrix. Thematic analysis was undertaken by LT, in consultation with TR and RM. A gradual process of interrogating similarities and differences in the data using the framework matrix occurred. Themes and sub-themes relating to the research question were identified. Preliminary findings were reviewed by LT, TR and RM.

### Member checking

Member checking activities were undertaken in December 2020 in order to establish credibility of findings. This involved emailing participants a summary of emerging themes and sub-themes, along with illustrative quotes to elicit participant feedback. Participants confirmed that the emerging themes accorded with their perspectives.

### Rigour

Rigour in qualitative research can be checked against four criteria: credibility, transferability, dependability, and confirmability [24]. Approaches used by the authors to ensure study rigour include: data triangulation through comparing individual viewpoints and experiences of the ARRT team against those of participants external to the ARRT team, and through consulting organisational documents describing the ARRT service [19, 25]; investigator triangulation through dual coding of data by a male and female researcher of different backgrounds and experiences [26]; the undertaking of member checking activities; reporting descriptive data relating to the study setting and context; and utilising the Consolidated Criteria for Reporting Qualitative Studies [27] in manuscript preparation.

## Results

A total of six health professionals were interviewed, comprising three (50.0%) nursing professionals, two (33.3%) medical professionals, and one (16.7%) paramedic professional. Four (66.7%) participants were female and two (33.3%) were male. Female participants were employed as nursing and paramedic professionals and male participants were employed as medical professionals. Five (83.3%) participants were staff from the two hospitals operating within the ARRT service area, and one (16.7%) was employed by NSW Ambulance. Of the five hospital staff, three (60.0%) were ARRT staff (two Clinical Nurse Consultants and a Geriatrician), one (20.0%) was an ED-based Clinical Nurse Specialist working specifically with older people who present to the ED, and one (20.0%) was a Clinical Director and Geriatrician. When asked how they felt about their job as a whole, two (66.7%) ARRT staff reported feeling extremely satisfied. One (33.3%) ARRT staff reported feeling extremely satisfied pre-COVID-19 and moderately satisfied post-COVID-19.

Analysis of interviews identified five overarching themes affecting ARRT utilisation in the care of acutely unwell RACF residents: (1) resident care need factors; (2) family factors; (3) enabling factors; (4) barriers; and (5) adaptability and responsiveness (Table 2). Sub-themes within themes were also identified.

**Table 2:** Overview of interview themes and sub-themes with illustrative quotes from health professionals

Theme	Sub-theme	Illustrative quotes	
1. Resident care need factors	1.1 Residents with cognitive impairment	*“The type of patient [who would benefit most from ARRT] would be someone who’s frail, who has dementia. That would be an example of condition where it’s better for them not to be in hospital if you can avoid it.”* (G1) Geriatrician	*[ARRT is able to give a resident with delirium] treatment that they would get in hospital, in a much more conducive environment to their actual health and well-being. They’re definitely reducing the adverse effects that they would be exposed in hospital.”* (CNC1) ARRT Clinical Nurse Consultant
	1.2 Residents at the end of life	*“If they’re palliative, if they are dying, they can be cared for - in the majority of facilities - they can be cared for at the facility.”* (CNC2) ARRT Clinical Nurse Consultant	*“We liaised with a palliative care team that hadn’t spoken to her for a few months. And we liaised with ARRT and we did a video conference. And we ended up providing care for her at home that then she was able to pass away after we’d implemented care. Which was amazing and it’s exactly what she wanted.”* (ECP) Extended Care Paramedic
2. Family factors	2.1 Family needs and expectations	*“Families, I think, still see hospital is this gold standard of care, and it is. But I think it’s very hard sometimes to explain to families that [for] their loved ones … the outcome of the treatment of going into hospital may not necessarily change the position or change the condition for their family - for their loved ones. So it’s managing those expectations.”* (CNC1) ARRT Clinical Nurse Consultant	*“There are benefits [of RACF-based management of residents] to the family. The patients kept where they are [and] the family can still visit, can be involved in their care.”* (G1) Geriatrician
3. Enabling factors	3.1 Rapid geriatric outreach service	*“[ARRT has the] ability to provide medical care outside of the hospital, and that includes comprehensive geriatric assessments. That includes provision of certain treatments. So things like antibiotics, fluids, ability to do blood testing, ability to do things like diagnostics. Things like ECGS, bladder scanners, mobile X-ray.”* (G1) Geriatrician	*We’re doing lots of acute assessments of patients … assessing if they’ve been unwell, assessing if they’ve got any sort of major things that we can reverse, but ultimately trying to keep them in the … aged care facility as well … Trying to treat them or apply sort of acute management outside of hospital.”* (CNC1) ARRT Clinical Nurse Consultant
	3.2 Coordination of care	*“I like the fact that AART can refer on to the other teams. So you’ve got the aged care, you’ve got APAC [Acute Post-Acute Care], you’ve got palliative care. And they could even arrange sort of direct admissions and bypass ED as well, so you don’t want necessarily these people to be sitting in emergency.”* (ECP) Extended Care Paramedic	*When I get the phone call from ARRT … I can just basically lookout for the patient as soon as they get on the screen. We can then kind of grab the medical team and say “Hey, let’s quickly get someone on it”, you know, get them seen quickly, get them their scans happening, and then try and get them out quickly. So if someone didn’t jump on board, they may sit in the ambo bay for - I don’t know how many hours … I do think that we can fast track them through [ED] to a degree.”* (CNS) ED-based Clinical Nurse Specialist
	3.3 Telehealth	*“We’re doing so many video conferences - that our capacities, it’s allowed our capacity to build up because you don’t need to go and do the reviews on site. Two reviews this morning already.”* (CNC2) ARRT Clinical Nurse Consultant	*You know, within 5 to 10 min we can start up a video conference with someone in an age care facility.”* (G1) Geriatrician 1
	3.4 Relevant skills within the ARRT team	*“We just work collaboratively. I think that is key to how the team actually works.”* (CNC2) ARRT Clinical Nurse Consultant	*“[ARRT] staff need to be relatively independent and relatively senior and experienced and able to deal flexibly with problems. I think those would be the core components, senior medical, senior nursing and a flexible approach to management.”* (G1) Geriatrician
	3.5 Relationships with other services	*“Develop good relationships with your aged care facilities and your GP’s that are working within them … it just opens up the pathway of referral. Developing pathways from NSW Ambulance into teams like the ARRT service, really developing good relationships with them as well, really opens up the ability to … intercept ambulances.”* (CNC1) ARRT Clinical Nurse Consultant	*“I personally think they’re amazing. It’s a really good service. It ticks the boxes for getting the right patient, the right care, in the right place. And they’re so easy to liaise with, and yeah just fantastic.”* (ECP) Extended Care Paramedic
	3.6 Capability building of RACF staff	*“While [reducing unnecessary hospital admissions is] our primary goal, our secondary goal, which is also very important, is educating, upskilling and providing support for aged care facility staff … with a similar goal in mind.”* (G1) Geriatrician 1	*“Building up the capacity within the nursing staff. So that they can recognise deterioration early, that they feel more competent and confident they can manage more acutely unwell people.”* (CNC2) ARRT Clinical Nurse Consultant
4. Barriers	4.1 Team size	*“Lately we’re overwhelmed with activity … We’re also working on coronavirus planning for nursing homes, which is entirely separate to our usual business. So time, inadequate numbers of staff. Although we’re trying to address that too. We were actually recruiting a lot of people recently.”* (G1) Geriatrician	*“We’re a small team. It would be great to have a bigger team. Something that we - and I guess that’s another barrier, is if we had more geriatricians, more staff, more expertise in the field, Allied Health that worked directly with us, we might be able to achieve a lot of different things.”* (CNC1) ARRT Clinical Nurse Consultant
	4.2 Hours of operation	*“Extended hours and weekends covered would be great... A lot of the time health issues arise, it’s not on a Monday-Friday 9–5.”* (ECP) Extended Care Paramedic	*“A lot of residential aged care patients do come in [to the ED] after hours, because they haven’t got services like aged care rapid response after hours... [They] do tend to come quite regularly after hours, and we don’t have that support then.”* (CNS) ED-based Clinical Nurse Specialist
5. Adaptability and responsiveness	5.1 The COVID-19 pandemic	*“Coronavirus has probably improved [communication between the hospital and RACFs] too. Because we’re increasing talking to the nursing home. We’re actually having face to face sessions with them. Trying to upskill them, and I guess all that raises awareness of what we’re doing.”* (G1) Geriatrician	*“[The hospital is] much more engaged and [RACFs} are much more engaged than they were six months ago, before the COVID. And particularly the last four months when the realisation of the devastating impact that that disease might have. There’s much more communication, and we’re out there training them on PPE, and infection control measures, right now. So that, in that very focused way we’re much more engaged together. Prior to that, it was spotty. Some places did well, and others, we hardly made much penetration in to at all.”* (G2) Geriatrician

*RACF* Residential aged care facility

### Theme 1—resident care need factors

Factors relating to resident care needs were the basis for considering whether specific resident health conditions were likely to benefit from ARRT intervention versus hospital treatment. When considering the risks and benefits of hospital transfer for unwell older RACF residents, participants recognised potential care complications for residents admitted to hospital. These care complications included delirium, falls and medication errors.

#### Sub-theme 1.1— residents with cognitive impairment

Five participants acknowledged that residents with cognitive impairment, such as delirium or dementia, were at risk of poor outcomes when transferred to hospital services. The ARRT geriatrician and CNCs considered the RACF a suitable environment for the management of residents with cognitive impairment.

*The type of patient [who would benefit most from ARRT] would be someone who's frail, who has dementia. That would be an example of condition where it's better for them not to be in hospital if you can avoid it.* (G1) Geriatrician

#### Sub-theme 1.2— residents at the end of life

All participants emphasised the identification of, and advocacy for, end of life care needs and the use of advance care directives as an important factor in preventing unnecessary ED transfers and hospital admissions. One participant described how a lack of RACF staff experience can lead to unnecessary hospital transfer for residents.

*And it's, it is very sad for people with dementia and things like that. [RACF staff] don't really understand the progression of dementia. They don't understand what end of life might look for these people, and how long that may progress for them. So they're kind of, I guess, sending people in unnecessarily, or you know, not instigating certain levels of care because of their lack of experience.* (CNC1) ARRT Clinical Nurse Consultant

As with cognitive impairment, the RACF was considered a suitable environment for residents at the end of life.

### Theme 2—family factors

All participants discussed the family of unwell residents as an important consideration in care planning and decisions. Understanding and navigating family needs and expectations was considered an essential, and at times complex, part of the health professional’s role.

#### Sub-theme 2.1—family needs and expectations

Four participants described the potential for tension between providing RACF-based treatment for the resident and respecting the family’s wishes for hospital treatment.

*Families, I think, still see hospital is this gold standard of care, and it is. But I think it's very hard sometimes to explain to families that [for] their loved ones … the outcome of the treatment of going into hospital may not necessarily change the position or change the condition for their family - for their loved ones. So, it's managing those expectations.* (CNC1) ARRT Clinical Nurse Consultant

One participant described the potential for conflict between the resident’s care preferences and the family’s care preferences, and how they can navigate this conflict with interpersonal communication.

*A lot of the times we’ll go to [RACFs], they've got an advance care plan, but the family will try and override that … At the end of the day, the patient’s rights are there. But if they're not able to communicate those anymore. It's the ability of the paramedic to communicate well with the family, I think. Get them on board, discuss with them what really they want happening, adverse to what they think should happen. And you can get the GP onboard as well and have a little chat, and I think, allay the fears. Cause I think a lot of the time people just think, oh just take them to hospital, they'll be OK. But at the end of the day, the condition probably isn't going to change.* (ECP) Extended Care Paramedic

Three participants viewed providing geriatric outreach services to RACFs as potentially benefitting families as well as residents.

*There are benefits [of RACF-based management of residents] to the family. The patients kept where they are [and] the family can still visit, can be involved in their care.* (G1) Geriatrician

### Theme 3—enabling factors

Enabling factors related to ARRT’s service elements. Notably, this included the provision of rapid geriatric outreach to RACFs, the coordination of care between services and providers, the utilisation of telehealth, relevant skills within the ARRT team, ARRT’s relationship with other services, and providing capability building opportunities for RACF staff.

#### Sub-theme 3.1—rapid geriatric outreach service

ARRT was noted for its ability to provide acute care outside of a hospital environment.

*[ARRT has the] ability to provide medical care outside of the hospital, and that includes comprehensive geriatric assessments. That includes provision of certain treatments. So things like antibiotics, fluids, ability to do blood testing, ability to do things like diagnostics. Things like ECGS, bladder scanners, mobile X-ray.* (G1) Geriatrician

#### Sub-theme 3.2—coordination of care

In addition to providing outreach services to RACFs, ARRT can facilitate the fast tracking of residents through the ED via the ED-based Clinical Nurse Specialist, as well as refer residents to other specialities e.g. Allied Health.

*When I get the phone call from ARRT … I can just basically lookout for the patient as soon as they get on the screen. We can then kind of grab the medical team and say “Hey, let's quickly get someone on it”, you know, get them seen quickly, get them their scans happening, and then try and get them out quickly. So if someone didn't jump on board, they may sit in the [ambulance] bay for - I don't know how many hours … I do think that we can fast track them through [ED] to a degree*. (CNS) ED-based Clinical Nurse Specialist

#### Sub-theme 3.3—telehealth

In July 2019, ARRT piloted the use of telehealth in five RACFs. Telehealth has since expanded to include all RACFs in the local service area. The use of telehealth was noted to greatly increase the ARRT team’s capacity and response time.

*We’re doing so many video conferences … it’s allowed our capacity to build up because you don't need to go and do the reviews on site. Two [resident] reviews this morning already.* (CNC2) ARRT Clinical Nurse Consultant

#### Sub-theme 3.4— relevant skills within the ARRT team

The composition of the ARRT team was noted by participants to be a key enabler of the ARRT service.

*I don't think ARRT would run without medical support. I think they need that, and relatively senior medical support. Similarly, I think that the, it wouldn't run without nursing, without a nursing team. It might, but it would be a much smaller service and less capable … I think all of our staff need to be relatively independent and relatively senior and experienced and able to deal flexibly with problems. I think those would be the core components [of ARRT], senior medical, senior nursing and a flexible approach to management.* (G1) Geriatrician

#### Sub-theme 3.5—relationships with other services

The development of good relationships with other services and providers was seen by the ARRT team to encourage referral to ARRT. This sentiment was echoed by participants external to the ARRT team.

*I personally think they're amazing. It’s a really good service. It ticks the boxes for getting the right patient, the right care, in the right place. And they're so easy to liaise with, and yeah just fantastic.* (ECP) Extended Care Paramedic

#### Sub-theme 3.6—capability building of RACF staff

Capability building of RACF staff is considered an important function of ARRT. Along with providing outreach services, capability building was perceived as helpful in reducing avoidable hospital admissions through empowering RACF staff to more effectively manage acutely unwell residents.

*Building up the capacity within the nursing staff. So that they can recognise deterioration early, that they feel more competent and confident they can manage more acutely unwell people.* (CNC2) ARRT Clinical Nurse Consultant

### Theme 4—barriers

Barriers to the utilisation of ARRT related to team size and the lack of support after hours and on weekends.

#### Sub-theme 4.1—team size

The small number of staff employed by ARRT was seen to create some restriction in terms of achievement. This was particularly amplified by the COVID-19 pandemic.

*We’re a small team. It would be great to have a bigger team. Something that we - and I guess that’s another barrier, is if we had more geriatricians, more staff, more expertise in the field, Allied Health that worked directly with us, we might be able to achieve a lot of different things.* (CNC1) ARRT Clinical Nurse Consultant.

#### Sub-theme 4.2—hours of operation

Health professionals external to the ARRT team expressed a desire for ARRT to extend its coverage to after hours and weekends.

*A lot of residential aged care patients do come in [to the ED] after hours, because they haven't got services like aged care rapid response after hours... [They] do tend to come quite regularly after hours, and we don't have that support then.* (CNS) ED-based Clinical Nurse Specialist

### *Theme 5—*adaptability and responsiveness

ARRT’s ability to adapt to changing circumstances was discussed in the context of the COVID-19 pandemic.

#### Sub-theme 5.1—the COVID-19 pandemic

The COVID-19 pandemic saw an increase in ARRT’s activity. In February/March 2020, the use of telehealth rapidly expanded from five pilot RACF sites to all 43 local RACFs within the ARRT service area.

*Since COVID hit - I really notice the difference in what we can achieve, how we will be utilising teleconferencing, and it really does change, I guess, your ability to be quick and rapid for your patient. You just understand it a whole lot more, and you're not relying so much on what actually someone’s telling you or how panicked, you know? People do tend to panic if the patient’s unwell. You're not necessarily getting a very clear picture. Whereas, if you can see them, and you can ask them questions, you can assess them over the phone. We can do very simple assessments with aged care facility staff … It's definitely been a crucial change in what we do.* (CNC2) ARRT Clinical Nurse Consultant

In response to the COVID 19 pandemic, the capacity building component of ARRT services expanded to cover outbreak management preparation and training of RACF staff. Participants reflected that communication and partnerships between the hospital and local RACFs was enhanced post-COVID-19

*[In addition to our normal activities] we have all the coronavirus stuff … face-to-face meetings, talking through outbreak management plans, and actually doing visits where we go out and site visit and work out if they’ll manage if there’s a coronavirus outbreak there. So there's a lot of upskilling going on at the moment.* (G1) Geriatrician

*[The hospital is] much more engaged and [RACFs] are much more engaged than they were six months ago, before the COVID … There's much more communication, and we're out there training them on PPE, and infection control measures, right now. So that, in that very focused way we’re much more engaged together. Prior to that, it was spotty. Some places did well, and others, we hardly made much penetration in to at all* (G2) Geriatrician

Funding was sought to expand the ARRT team. As of October 2020, when interviews were conducted, ARRT was in the process of hiring additional medical, nursing and allied health staff.

*You know we're talking about our service, how good we are, we're giving them our figures and they're enhancing our staff at the moment because of COVID. We aren't a team that sat back and just let it happen. It's like no, we need this, we need that, we need this person in the position, we need this person in that position. We're not gonna just accept what you give us.* (CNC2) ARRT Clinical Nurse Consultant

## Discussion

This study identified factors affecting ARRT utilisation in the care of acutely unwell RACF residents, as perceived by health professionals. Resident care needs and family needs and expectations were recognised as contributing factors for resident care decisions and care plans. ARRT’s service elements were recognised as enabling factors for hospital avoidance, as was ARRT’s adaptability and responsiveness to the COVID-19 pandemic.

RACF residents with cognitive impairment were identified by participants as more suitable for RACF-based treatment than hospital-based treatment. Notably, an analysis of hospital service use trajectories of RACF residents reviewed by ARRT found residents living with dementia were more likely to be low hospital service users, compared to higher hospital service users [28]. Similarly, international studies have identified lower hospital service use in RACF residents with dementia, compared to residents without dementia [29–31]. There is evidence to suggest that care for residents with dementia may prioritise end of life care strategies, rather than active treatment, which may explain lower hospital service use by this cohort [32].

Family needs and expectations were recognised as an important and complex component of resident care decisions and planning, particularly when it came to residents at the end of life. Likewise, a qualitative study exploring factors influencing the transfer of Australian RACF residents to the ED found it was common for RACF staff to feel pressured by a resident’s family to seek active treatment for residents at the end of life, and therefore they were more likely to transfer the resident to the ED [15]. Furthermore, an evaluation of an Australian aged care nurse practitioner-based hospital avoidance service found that nurse practitioner consultation with the unwell RACF resident and their family was a major contributor to resident and family satisfaction, as well as hospital avoidance [33]. The use of advance care directives were noted as an important factor in preventing unnecessary ED transfers and hospital admissions. Comparably, two quasi-experimental studies investigating the use of advance care planning tools for RACF residents found reduced hospital admissions [34, 35].

.ARRT’s key service elements are providing rapid geriatric outreach to RACFs, coordinating care between health services and aged care providers, the utilisation of telehealth, skills within the ARRT team, forming relationships with other services, and capability building of RACF staff. Previously, an integrative review investigating models of care that avoid or improve transitions to hospital services for RACF residents found that whilst there is great heterogeneity amongst models, there is some evidence that the provision of geriatric outreach services to RACFs and the capability building of RACF staff may prevent some types of hospital admissions [11]. The review also reported a case study assessing the feasibility of telemedicine in providing geriatric services to RACF residents and found telemedicine may result in increased productivity and savings. Furthermore, evidence from a quasi-experimental evaluation of health outcomes of aged care residents that use ARRT services suggests that ARRT has a high success rate of RACF-based treatment of residents, as well as in reducing hospital length of stay and in hospital treatment costs compared to residents receiving usual care [12].

The results of the quasi-experimental evaluation on their own may suggest further evidence supporting the provision of geriatric outreach services to RACFs and capability building of RACF staff by hospital avoidance programs. At the same time, it should be noted that participants of the current study emphasised relevant skills within the ARRT team and the quality of relationships with other services as enablers of ARRT’s success. Similarly, a structure and process evaluation of an Australian hospital avoidance program found that collaboration and effective communication and referral facilitates the provision of geriatric outreach services to RACF residents [36]. Additionally, a qualitative evaluation of stakeholder perspectives on transitions of RACF residents to EDs in two Canadian provinces found that successful transitions were influenced by effective communication and positive relationships with families and other providers [37]. Furthermore, a qualitative study exploring RACF staff and GP perspectives on RACF-based management and ED transfer of unwell residents in NSW, Australia stated that positive care transitions for unwell residents could be facilitated by developing positive relationships; and communication and collaboration between RACFs, ambulance, EDs and GPs [16]. Finally, an interview study investigating decision making around hospital transfer and/or referral to a geriatric outreach service in Melbourne, Australia discovered that the availability of timely and appropriately skilled medical and nursing staff to RACFs greatly influenced care decisions [38]. Therefore, when designing and implementing hospital avoidance models of care, consideration must be given to team skills, interpersonal communication, and relationship building with families and other health care providers. This underscores the importance of conducting mixed methods evaluation of complex interventions, with qualitative studies able to provide key context factors to enhance quantitative findings.

The COVID-19 pandemic has disrupted clinical and aged care in RACFs, necessitating quick adaption and response by health care teams to ensure they’re meeting the care needs of this especially vulnerable population [39]. ARRT has demonstrated adaptability and responsiveness to the COVID-19 pandemic. Prior to the pandemic, ARRT piloted the use of telehealth in five local RACFs. In response to COVID-19, ARRT expanded telehealth to include all RACFs in the local service area. The introduction and utilisation of teleconferencing was seen to enhance the rapidness of ARRT’s assessment and response to unwell residents. In addition to the expansion of telehealth, ARRT was involved in coronavirus planning and outbreak management with RACFs. This extra activity overwhelmed the ARRT team, leading them to seek additional funding and recruit additional medical, nursing and allied health staff. Similarly, a rapid exploration of emerging evidence regarding the care of older people during COVID-19 found evidence that health and social care teams can transform very rapidly in response to COVID-19, with goodwill driving much of this change. Team transformation included the use of telehealth, increased trust and collaboration within teams; and teams feeling empowered to drive change at a local level [39]. Given the paucity of evidence concerning hospital-based versus RACF-based treatment of older RACF residents with COVID-19 [40], the current study provides further evidence of a health team transforming in response to a pandemic.

### Limitations

The geographic coverage of ARRT and the hours that ARRT is available, as well as the healthcare system it operates within, limit the generalisability of the current study’s findings. Local healthcare system and population differences should be considered when interpreting the current study’s findings. It was the intention of the current study to incorporate the views of RACF staff and General Practitioners. However, recruitment of interview participants occurred after the COVID-19 lockdown in Australia. Although attempts were made to recruit RACF staff and General Practitioners, these attempts were unsuccessful. Nonetheless, the current study’s findings concerning the promotion of communication and relationship building between services is supported by a study solely utilising the perspective of RACF staff and GPs in a similar setting. Future research should incorporate the views of RACF staff, General Practitioners, and Paramedics other than Extended Care Paramedics, as well as the views of RACF residents and family members.

## Conclusion

The current study has identified various factors impacting hospital avoidance program utilisation in the care of acutely unwell aged care residents. Notably, this includes providing access to timely and skilled care, promoting interpersonal communication and relationships, and transformation in response to the COVID-19 pandemic. The current study may provide additional context to existing quantitative evaluations of hospital avoidance programs, as well informing the design of future hospital avoidance programs.

## Supplementary Information

**Additional file 1.**

## Data Availability

The datasets used and/or analysed during the current study are available from the corresponding author on reasonable request.

## Abbreviations

ARRT: Aged Care Rapid Response Team
ED: Emergency Department
NSLHD: Northern Sydney Local Health District
RACF: Residential Aged Care Facility
