# Supplementary material for: Factors impacting hospital avoidance program utilisation in the care of acutely unwell residential aged care facility residents
Source: BMC Health Serv Res. 2021 Jun 24;21:599. doi: 10.1186/s12913-021-06575-1 (PMC8221986; doi:10.1186/s12913-021-06575-1)
Supplement: Supplementary file 1 — Additional file 1. [file 12913_2021_6575_MOESM1_ESM.docx]

**Factors impacting hospital avoidance program utilisation in the care of acutely unwell residential aged care facility residents**

Luke Testa*^1^; Tayhla Ryder^1^; Jeffrey Braithwaite^1^; Rebecca J Mitchell^1^

Authors’ Institutional affiliation(s):

^1^ Australian Institute of Health Innovation, Macquarie University, Level 6, 75 Talavera Road, Macquarie University NSW Australia 2109

* Corresponding author:

Mr Luke Testa; Level 6, 75 Talavera Road, Macquarie University NSW Australia 2109; luke.testa@mq.edu.au

**APPENDIX A. INTERVIEW GUIDES**

**Interview guide - ARRT staff**

1. Can you tell me about your role and relationship to ARRT?
2. How would you describe the purpose and goals of ARRT?
3. What effect do you believe ARRT has on hospital service use by residents?
4. What effect do you believe ARRT has on resident health outcomes?
5. What are the main features of ARRT that make it patient-centred?
6. How rapidly can residents access ARRT assistance when required?
7. Are there any formal guidelines for the management of residents within ARRT?
8. How well do you think the coordination of resident care between aged care facilities and the hospital is working?
9. Has any capacity building been conducted, as needed, to build the capabilities of aged care facility staff to assess and care for unwell residents?
10. How does comprehensive geriatric assessment form part of resident care provided by ARRT?
11. How does ARRT contribute to advanced care planning as part of resident care?
12. What are the main benefits/risks of treating unwell residents in the facility?
13. What are the main benefits/risks of hospital transfer?
14. What are the key factors that help you decide if a resident needs to be transferred to hospital, rather than be treated in the facility?
15. In your opinion, to what degree is care personalised to resident’s and their families’ feelings and preferences?
16. How would you describe the partnership between the hospital and aged care facilities in relation to resident care?
17. How would you describe communication between ARRT and other services in relation to the residents’ care plan?
18. In your view, would there be opportunities to improve the partnership and/or communication between hospitals and aged care facilities?
19. Taking everything into consideration, how do you feel about your job as a whole?’
    - 1=extremely dissatisfied, 2=moderately dissatisfied, 3=slightly dissatisfied; 4=neutral, 5=slightly satisfied, 6=moderately satisfied, 7 = extremely satisfied
    - Why have you given this rating?
20. Has there been much staff turnover in ARRT?
21. In your view, is there anything that can be changed about ARRT to improve the service?
22. Any other comments that you wish to make in relation to ARRT?

**Interview guide - Hospital staff**

1. Can you tell me about your role and relationship to ARRT?
2. How would you describe the purpose and goals of ARRT?
3. What effect do you believe ARRT has on hospital service use by residential aged care residents?
4. What effect do you believe ARRT has on resident health outcomes?
5. How well do you think the coordination of resident care between aged care facilities and the hospital is working?
6. What are the main benefits/risks of hospital transfer for residents?
7. In your opinion, to what degree is care personalised to resident’s and their families’ feelings and preferences?
8. How would you describe the partnership between the hospital and aged care facilities in relation to resident care?
9. How would you describe communication between ARRT and other services in relation to the residents’ care plan?
10. In your view, would there be opportunities to improve the partnership and/or communication between hospitals and aged care facilities?
11. Taking everything into consideration, how do you feel about your job as a whole?’
    - 1=extremely dissatisfied, 2=moderately dissatisfied, 3=slightly dissatisfied; 4=neutral, 5=slightly satisfied, 6=moderately satisfied, 7 = extremely satisfied
    - Why have you given this rating?
12. In your view, is there anything that can be changed about ARRT to improve the service?
13. Any other comments that you wish to make in relation to ARRT?

**Interview guide – Extended care paramedic**

1. Can you tell me about your role and relationship to ARRT?
2. How would you describe the purpose and goals of ARRT?
3. What effect do you believe ARRT has on hospital service use by residents?
4. What effect do you believe ARRT has on resident health outcomes?
5. How well do you think the coordination of resident care between aged care facilities and the hospital is working?
6. What are the main benefits/risks of treating unwell residents in the facility?
7. What are the main benefits/risks of hospital transfer?
8. What are the key factors that help you decide if a resident needs to be transferred to hospital, rather than be treated in the facility?
9. Taking everything into consideration, how do you feel about your job as a whole?’
   - 1=extremely dissatisfied, 2=moderately dissatisfied, 3=slightly dissatisfied; 4=neutral, 5=slightly satisfied, 6=moderately satisfied, 7 = extremely satisfied
   - Why have you given this rating?
10. In your view, is there anything that can be changed about ARRT to improve the service?
11. Any other comments that you wish to make in relation to ARRT?
